# Intergeneric Comparison of Staminal Trichomes in the Tribe Ipomoeeae (Convolvulaceae)

**DOI:** 10.3390/plants13152050

**Published:** 2024-07-25

**Authors:** Natthaphong Chitchak, Alyssa B. Stewart, Paweena Traiperm

**Affiliations:** Department of Plant Science, Faculty of Science, Mahidol University, Bangkok 10400, Thailand; natthaphong.chitchak@outlook.com (N.C.); alyssa.ste@mahidol.edu (A.B.S.)

**Keywords:** androecia, morning glory family, plant anatomy, plant micromorphology, systematics

## Abstract

Hairs or trichomes distributed on the base of filaments in the morning glory family, known as staminal trichomes, differ from those found on other plant parts and have been recognized for their taxonomic value for over a century. In this study, our aim was to investigate the appearance of staminal trichomes in the tribe Ipomoeeae Hall. f., a significant tribe within the family Convolvulaceae, and assess their taxonomic implications. Micromorphological examinations were conducted using clearing techniques on 73 taxa from seven out of eight genera within the tribe, and the data were analyzed using factor analysis of mixed data (FAMD). The results show that, among all examined taxa, only two species lacked staminal trichomes. Glandular staminal trichomes were the most common type observed, and they were the only type observed in *Ipomoea*, *Paralepistemon*, *Rivea*, *Stictocardia*, and most *Argyreia* species. Nonglandular staminal trichomes were restricted to *Astripomoea*, *Lepistemon*, and some *Argyreia* taxa. The glandular trichomes in *Ipomoea* exhibited the largest variation and overlapped with other glandular trichome-bearing genera. However, genera with nonglandular trichomes were readily distinguishable from each other. Both glandular and nonglandular trichomes were basically composed of a stalk and apical cell, yet they varied in shape, size, density, and distribution pattern. This study provides a detailed examination and application of microscale features, emphasizing the significance of micromorphology in plant taxonomy.

## 1. Introduction

Floral elaboration appears in many shapes and forms, whether on sepals, petals, bracts, or other organs. Such floral evolution primarily serves a reproductive purpose by increasing pollinator attraction [1,2]. In the Convolvulaceae, although the corolla is relatively simple, the stamens are typically elaborated with trichomes along the base (where they attach to the corolla tube), which are called staminal trichomes [3,4,5]. These staminal trichomes were indeed confirmed to accumulate substances related to pollinator attraction, such as terpenoids [6,7]. Moreover, staminal trichomes in the Convolvulaceae have also long been known for their taxonomic value, especially in species identification and delimitation [8,9,10,11,12,13,14,15,16,17,18,19]. Most taxonomic work utilizing staminal trichomes has been conducted in the tribe Ipomoeeae Hall. f., a major tribe in the family [8,9,10,11,12,13,14,15,16,17,18,19]. For example, Ooststroom and Hoogland often used characters of staminal trichomes in the identification key of Malaysian *Argyreia* Lour. and *Ipomoea* L. and also employed them to resolve taxonomic misunderstandings between *Argyreia* species [8,9,10,11,12,13].

Similar to other taxonomically valuable microscale structures, such as leaf micromorphology and anatomy, observation of the fine details of staminal trichomes requires the use of microscopic techniques [20,21,22,23,24,25]. However, one factor that makes studying staminal trichomes rather more difficult is that they are hidden inside the flowers, making it almost impossible to obtain data from dry herbarium specimens. Therefore, in previous studies, information about these trichomes is typically superficial or neglected. For instance, reports often only mention the presence or absence of trichomes or describe their texture using modifiers such as pubescent, pilose, or villose [12,14,26], with only a few recent cases providing detailed descriptions of the staminal trichomes [5,27,28,29]. Moreover, studies by Chitchak et al. [27] and Staples et al. [30] have pointed out that staminal trichomes can vary in terms of size, structure, abundance, and distribution pattern. However, a comprehensive assessment across genera within any of the lineages within the Convolvulaceae has never been conducted.

Tribe Ipomoeeae currently encompasses nearly 1000 spiny pollen species classified into eight genera, i.e., *Argyreia* (including *Blinkworthia* Choisy), *Astripomoea* A. Meeuse, *Ipomoea* (including *Turbina* Raf.), *Lepistemon* Blume, *Lepistemonopsis* Dammer, *Paralepistemon* Lejoly & Lisowski, *Rivea* Choisy, and *Stictocardia* Hallier f. They are mainly distributed in the tropical Old World, except for *Ipomoea*, which is distributed worldwide [31,32,33]. However, genera within this tribe have recently undergone reclassification based on phylogenetic approaches, suggesting that they should be nested within a single genus, *Ipomoea* [34,35,36]. Yet, this suggestion has not been widely adopted due to the consequences of significant nomenclatural reduction [33,37]. Although these segregate genera are well-established, further studies in phylogenetics, phenetics, ecology, and other aspects are required to shed light on their relationships and help enhance the robustness of their taxonomic boundaries.

Therefore, in this study, we aimed to explore the variation in staminal trichomes across genera within the tribe Ipomoeeae and assess their taxonomic implications. Plant materials from spirit or dry specimens of 73 taxa (163 accessions) from seven of the eight genera were used in this study. Specimens of the African monospecific *Lepistemonopsis* were not able to be examined due to their rarity in collections. Staminal trichomes were anatomically examined for details concerning their type, shape, size, density, and distribution pattern. These characters were then analyzed using factor analysis of mixed data (FAMD) to visualize the variation among genera and identify potentially important characters for taxonomy.

## 2. Materials and Methods

### 2.1. Plant Materials

Flowers of *Argyreia* and *Ipomoea* were mainly collected from their natural habitats in Thailand, with some from cultivation. Whole fully opened flowers were preserved in 70% ethyl alcohol with a few drops of glycerol prior to micromorphological investigation. Species identification was conducted using the *Flora of Thailand* [26] and other related literature, such as the *Flora of China* [14], the *Flore Générale de l’Indochine* [38], and *Convolvulaceae: Flora of Cambodia, Laos and Vietnam* [39]. Voucher specimens were prepared using the standard method for plant taxonomy [40] and were deposited at the Forest Herbarium (BKF) or Suan Luang Rama IX Herbarium, Bangkok, Thailand. Additionally, spirit specimens of *A. hookeri* C.B. Clarke and *A. rubicunda* Choisy were collected from Indonesia and Myanmar, respectively, by P. Rattanakrajang, while *S. tiliifolia* (Desr.) Hallier f. was collected from Hawaii, USA by G. W. Staples. Flowers of other species and genera that are uncommon in Thailand, such as *Astripomoea* and *Paralepistemon*, were obtained from dry specimens kept at the herbarium at the Royal Botanic Gardens, Kew, UK (K). For dry specimens, flowers were first rehydrated by soaking them in soapy water overnight and were then transferred to 70% ethyl alcohol with a few drops of glycerol. Specimen details of all the species used in this study, including specimen type, collector no., and country collected, are listed in Table S1. In total, we used 73 taxa (163 accessions, with 1–4 accessions per taxon): *Argyreia* (35 taxa, 81 accessions), *Astripomoea* (7 taxa, 18 accessions), *Ipomoea* (21 taxa, 43 accessions), *Lepistemon* (4 taxa, 7 accessions), *Paralepistemon* (1 taxon, 3 accessions), *Rivea* (2 taxa, 4 accessions), and *Stictocardia* (3 taxa, 7 accessions).

### 2.2. Micromorphological Investigation

The lower part of corolla (including the base of epipetalous stamens) was excised in order to examine the entire distribution of staminal trichomes. These samples were then cleared using an alkaline solution of 10% potassium hydroxide for 5–30 min, depending on the size of the sample, to remove dark substances from the tissue. Afterward, the samples were transferred to a bleaching solution of 10% Clorox for four hours to enhance their transparency. Following clearing treatment, the materials were rinsed thoroughly and then left in tap water overnight to further remove any remaining clearing solution. Cleared samples were stained with 0.1% Toluidine blue O [41] and then temporarily mounted in concave slides with tap water. The slides were immediately observed under light microscope (Olympus CX21, New York Microscope Company, Hicksville, NY, USA) equipped with a Sony α6400 (Sony, Tokyo, Japan) digital camera and visualized using ToupView software (ToupTek, Hangzhou, China). The following staminal trichome characters were observed and measured: presence of trichomes, distribution pattern, entire trichome length, stalk width, apical cell shape, apical cell length and width, and trichome density. Data were collected from 1–3 replicates (flowers) per accession. Preliminary examination showed that the observed characteristics of the rehydrated dry materials were not significantly different from those of materials preserved in spirits.

### 2.3. Statistical Analyses

R version 4.3.1 [42] was used for all analyses. Our micromorphological investigation revealed that plant species could be categorized into three main groups: (i) species without staminal trichomes, (ii) species with only glandular staminal trichomes, and (iii) species with nonglandular staminal trichomes (either solely or in combination with glandular trichomes). Therefore, analyses were conducted for groups (ii) and (iii), and they were analyzed separately using characters listed in Tables S2 and S3. One-way ANOVA (function *aov* in package “stats”) was conducted to assess the statistical significance of quantitative characters among genera, and for significant characters, Tukey’s tests were used for post hoc analyses (function *emmeans* in package “emmeans”, visualized with function *cld* in package “multcomp”) [43]. Factor analysis of mixed data (FAMD) was conducted to visualize the variation among staminal trichomes in two-dimensional plots (function *PCAmix* in package “PCAmixdata”) [44]. Eigenvalues and character loadings were extracted from the FAMD. To identify high-loading characters (characters that strongly influence the distribution of data points on scatter plots), k-mean values (function *threshold* in package “mmand” and function *findthresh* in package “evir”) [45,46] were used to find the threshold of loading values among characters. All graphical visualizations of statistical analyses were generated using package “ggplot2” [47].

## 3. Results

### 3.1. Micromorphology of Staminal Trichomes in Tribe Ipomoeeae

**Figure 1:**
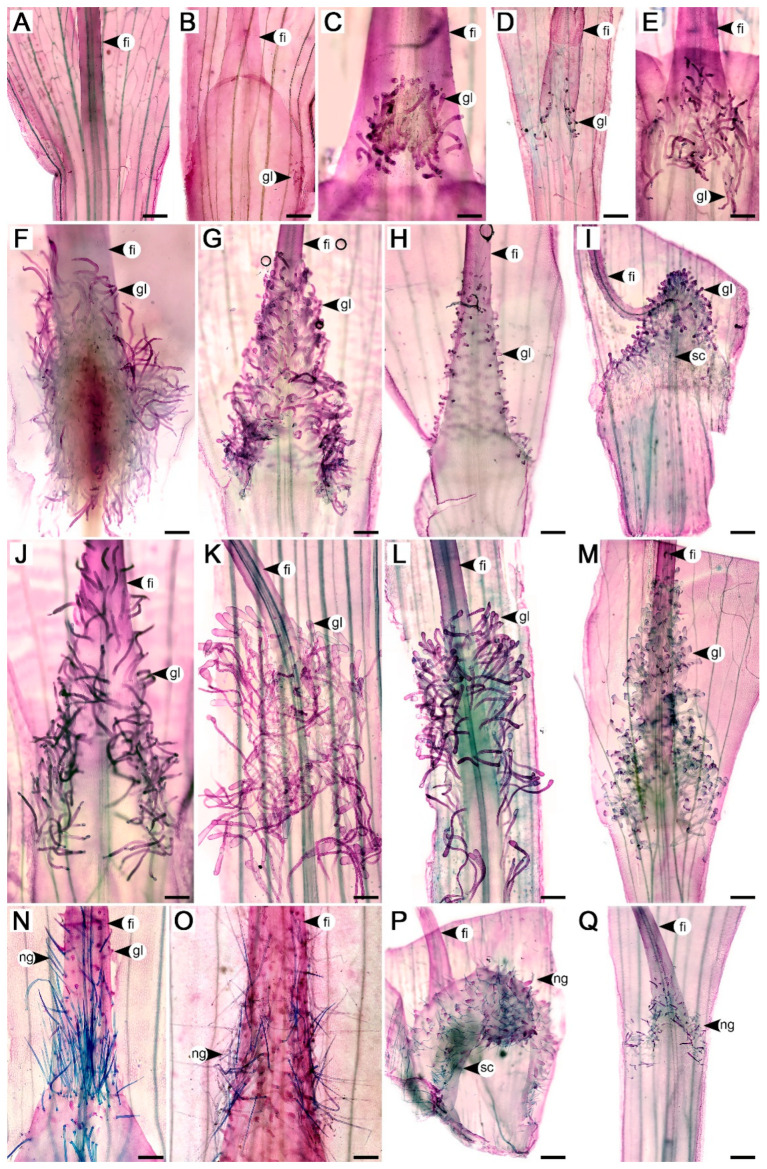
Filament bases of some species in the tribe Ipomoeeae: (**A**) *Ipomoea stenosiphon*, a species without staminal trichomes. (**B**) *Argyreia roseopurpurea*, the only species with clusters of glandular staminal trichomes along the lateral sides of filament bases. (**C**) *A. gyrobracteata*, a species with a small patch of glandular staminal trichomes on adaxial filaments. (**D**–**F**) species with glandular staminal trichomes distributed from adaxial to lateral sides of filament bases. (**D**) *I. pes-tigridis*. (**E**) *A. mollis.* (**F**) *Stictocardia tiliifolia*. (**G**–**M**) species with glandular staminal trichomes distributed on all sides of filament bases. (**G**) *I. pes-caprae*. (**H**) *A. lycioides*. (**I**) *Paralepistemon shirensis*. (**J**) *A. laotica*. (**K**) *Rivea ornata*. (**L**) *R. wightiana*. (**M**) *I. corymbosa*. (**N**–**P**) species with nonglandular staminal trichomes distributed on all sides of filament bases. (**N**) *A. collinsiae*. (**O**) *A. dokmaihom*. (**P**) *Lepistemon owariensis*. (**Q**) *Astripomoea malvacea* var. *malvacea*, a species with nonglandular staminal trichomes distributed on adaxial to lateral sides of filament bases. Abbreviations: fi, filament; gl, glandular staminal trichome; ng, nonglandular staminal trichome; sc, staminal scale. Scale bars = 500 μm.

Two species did not have trichomes at the base of staminal filaments, *Argyreia adpressa* (Choisy) Boerl. and *Ipomoea stenosiphon* Hallier f., and were categorized as group (i) (Figure 1A). The remaining species had staminal trichomes and were further categorized as species with only glandular staminal trichomes (group (ii); 55 taxa, accounting for 75% of all examined taxa) and species with nonglandular staminal trichomes (group (iii); 16 taxa, accounting for 22% of all examined taxa) (Figure 1). However, glandular trichomes were also found in some members of group (iii), with details provided in the following sections. Staminal trichome characters of all examined accessions are summarized in Table S4.

**Figure 2:**
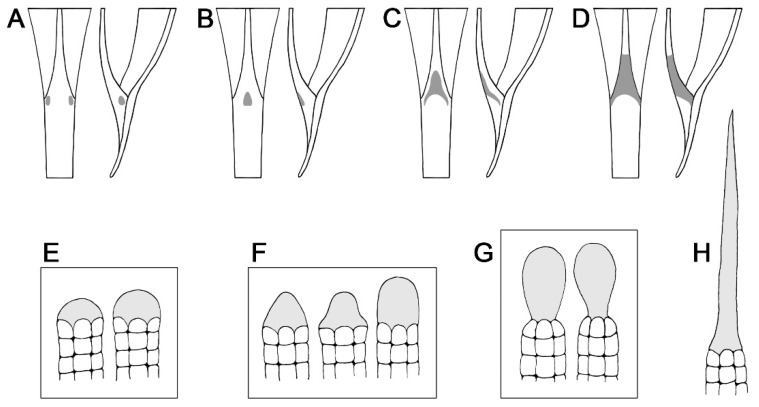
Illustrations of (**A**–**D**) staminal trichome distribution patterns and (**E**–**H**) general variations in apical cells. Panels (**A**–**D**) show the area of the flower excised for micromorphological investigation (base of corolla where filament bases attach to adaxial corolla surface; note: only one filament base is shown in illustrations) with areas occupied by staminal trichomes indicated in gray. (**A**) Staminal trichomes restricted to lateral sides of filament base. (**B**) Staminal trichomes appear as a patch on adaxial side of filament base. (**C**) Staminal trichomes distributed from adaxial to lateral sides of filament base. (**D**) Staminal trichomes found on all sides around filament base. (**E**) Glandular staminal trichomes with convex and globose apical cells. (**F**) Glandular staminal trichomes with rounded conical, bell-shaped, and rounded cylindrical apical cells. (**G**) Glandular staminal trichomes with obovoid and pyriform apical cells. (**H**) Simple nonglandular trichome.

**Figure 3:**
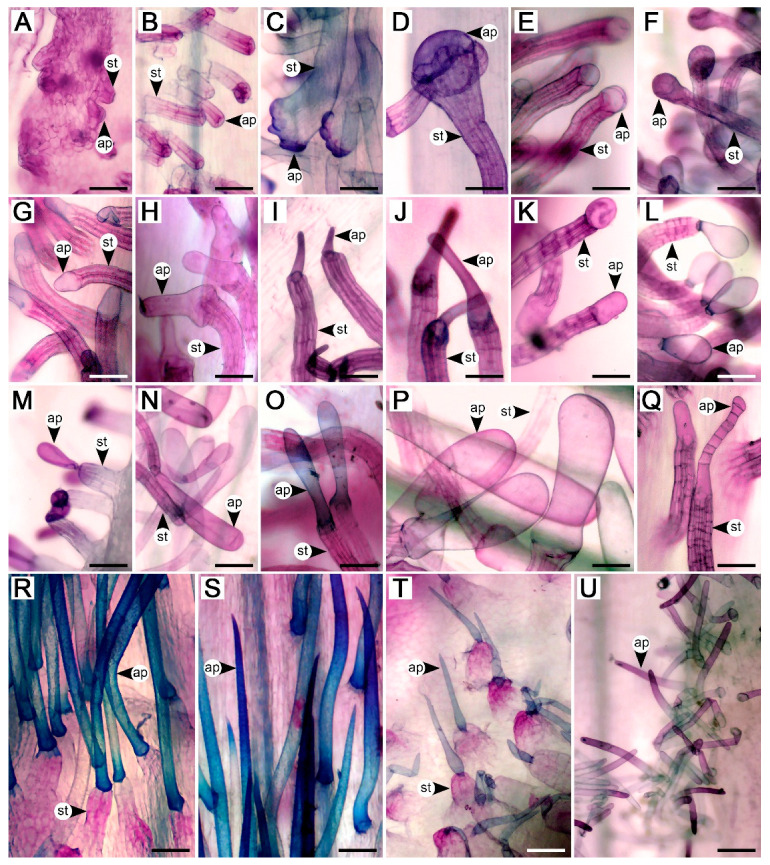
Photos taken via microscope of staminal trichomes from some species in the tribe Ipomoeeae: (**A**–**F**) Glandular staminal trichomes with convex to globose apical cells. (**A**) *Argyreia roseopurpurea*. (**B**) *Ipomoea quamoclit*. (**C**) *I. corymbosa*. (**D**) *I. alba*. (**E**) *A. confusa*. (**F**) *Paralepistemon shirensis*. (**G**–**K**) Glandular staminal trichomes with rounded conical, bell-shaped, and rounded cylindrical apical cells. (**G**) *A. capitiformis*. (**H**) *A. mekongensis*. (**I**) *I. indica*. (**J**) *A. thorelii*. (**K**) *A. roxburghii*. (**L**–**P**) Glandular staminal trichomes with obovoid and pyriform apical cells. (**L**) *I. pes-caprae*. (**M**) *A. lycioides*. (**N**) *I. obscura*. (**O**) *A. kerrii*. (**P**) *Rivea ornata*. (**Q**) Multicellular apical gland, a unique kind of glandular staminal trichome from *A. henryi*. (**R**–**T**) Nonglandular staminal trichomes with pointed tips. (**R**,**S**) *A. collinsiae*. (**T**) *Lepistemon owariensis*. (**U**) Nonglandular staminal trichomes with rounded tips from *Astripomoea malvacea* var. *malvacea*. Abbreviations: ap, apical cell; st, stalk. Scale bars = 100 μm.

**Figure 4:**
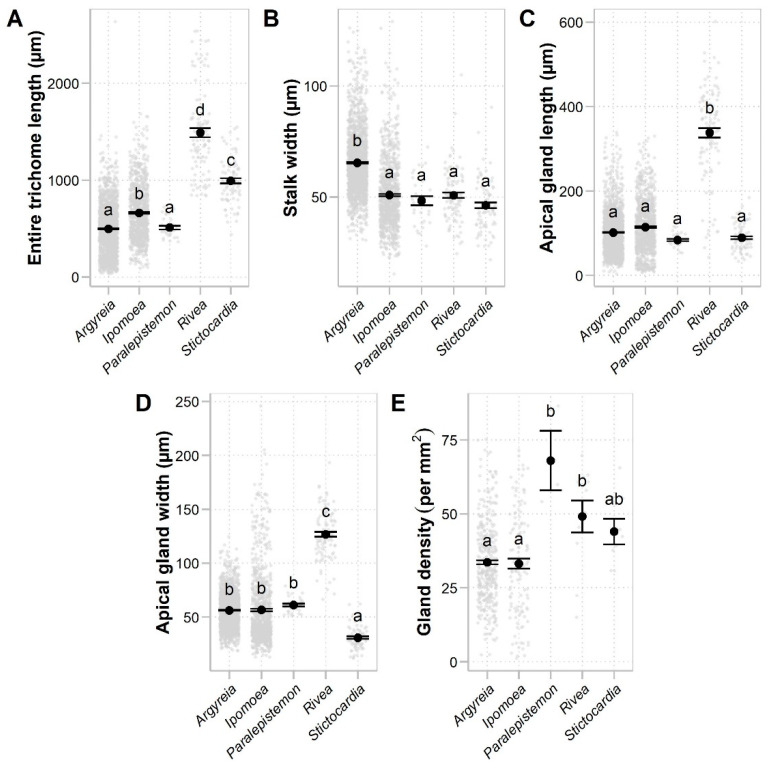
Comparisons of quantitative characters of staminal trichomes between genera with only glandular staminal trichomes (group ii): (**A**) Entire trichome length. (**B**) Stalk width. (**C**) Apical gland length. (**D**) Apical gland width. (**E**) Gland density. Black dots and error bars denote means and standard errors. Gray dots indicate raw data. Genera with different letters are significantly different (*p* < 0.05).

Staminal trichomes were typically distributed at the very base of the filaments with the center of distribution located along adaxial, lateral, or abaxial surfaces of the widest portion, close to where the filaments attach to the corolla. The parts of the filaments where they adjoined to each other usually lacked trichomes. They were either evenly distributed throughout the entire area (Figure 1G), or densely distributed in the center of the distribution (on the adaxial, lateral, or abaxial surface), and gradually became sparse toward the margins of their distribution (Figure 1H,J). For some species with narrow corolla tubes, such as *I. alba* L. and *Rivea wightiana* R.R. Mill, the trichomes extended to the adaxial corolla surface (Figure 1L). The distribution pattern of the staminal trichomes was highly consistent within species. There were four patterns found: (1) small clusters located along the lateral sides of staminal filaments (one species, *A. roseopurpurea* (Kerr) Ooststr.) (Figure 1B and Figure 2A), (2) a small patch restricted to the center of the adaxial side of filaments (two species, *A. breviscapa* (Kerr) Ooststr. and *A. gyrobracteata* Traiperm & Chitchak) (Figure 1C and Figure 2B), (3) a relatively broad area ranging from the center of the adaxial surface of filaments downward to both lateral sides (20 taxa, accounting for 28% of species with staminal trichomes present) (Figure 1D–F,Q and Figure 2C), and (4) covering all sides of the lower part of the filaments (48 taxa, accounting for 68% of all species with staminal trichomes present) (Figure 1G–P and Figure 2D).

Glandular staminal trichomes were found in *Argyreia*, *Ipomoea*, *Paralepistemon*, *Rivea*, and *Stictocardia*. The trichomes were composed of two parts, the stalk and apical gland, but the basal cells could not be differentiated from the stalk cells or regular epidermal cells. The stalk was composed of vertically stacked, cylindrical-shaped cells. The apical gland was typically unicellular (Figure 3A–P,R–U), except in *A. henryi* (Craib) Craib and *A. melvillei* (S. Moore) Staples, where trichomes with multicellular glands were scattered among those with unicellular glands (Figure 3Q). Within a species, we typically observed trichome apical glands with only a single shape or a group of related shapes, yet multiple shapes could be found within a genus (Table 1). They were categorized into three groups, grouping shapes that were often found together: (1) convex and globose apical glands (14 taxa, accounting for 23% of species with glandular staminal trichomes present) (Figure 2E and Figure 3A–F), (2) rounded conical, bell-shaped, and rounded cylindrical apical glands (27 taxa, accounting for 45% of species with glandular staminal trichomes present) (Figure 2F and Figure 3G–K), and (3) obovoid and pyriform apical glands (19 taxa, accounting for 32% of species with glandular staminal trichomes present) (Figure 2G and Figure 3L–P). Results of the ANOVA reveal that all measured variables (entire glandular trichome length, stalk width, apical gland length, apical gland width, and gland density) were significantly different among genera (Figure 4; Table 1).

**Figure 5:**
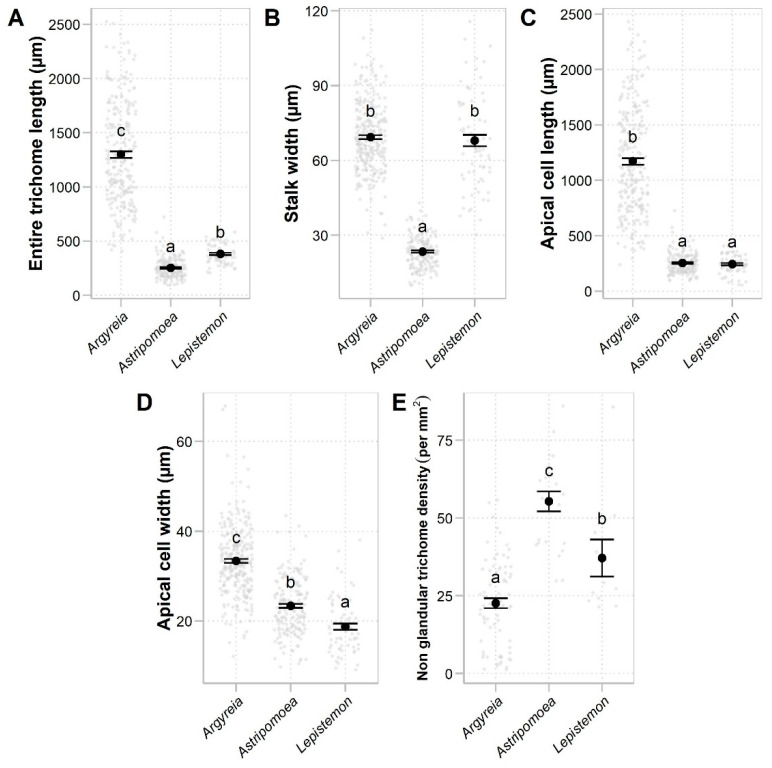
Comparisons of quantitative characters of staminal trichomes between genera with nonglandular staminal trichomes (group iii): (**A**) Entire trichome length. (**B**) Stalk width. (**C**) Apical cell length. (**D**) Apical cell width. (**E**) Nonglandular staminal trichome density. Black dots and error bars denote means and standard errors. Gray dots indicate raw data. Genera with different letters are significantly different (*p* < 0.05).

Nonglandular staminal trichomes were found in *Astripomoea*, *Lepistemon*, and five taxa of *Argyreia*. In *Argyreia*, glandular trichomes were sparsely distributed among nonglandular trichomes (Figure 1N), while staminal trichomes in *Astripomoea* and *Lepistemon* were purely nonglandular (Figure 1P,Q and Figure 3T,U). Similar to the glandular trichomes, nonglandular trichomes comprised two distinct parts, a multicellular stalk and an apical cell. The structure of the stalks of nonglandular trichomes was similar to those found in glandular trichomes. The apical cell was simple, unicellular, and slender, with a pointed tip in *Argyreia* and *Lepistemon* (Figure 1N–P and Figure 3R–T) but a rounded tip in *Astripomoea* (Figure 1Q and Figure 3U). Apical cells exhibiting splitting or branching were only rarely found in *A. suddeeana* Traiperm & Staples and *A. dokmaihom* Traiperm & Staples, respectively. Results of ANOVA reveal that all measured variables (entire nonglandular trichome length, stalk width, apical cell length, apical cell width, and nonglandular trichome density) were significantly different among genera (Figure 5; Table 2).

### 3.2. Factor Analysis of Mixed Data (FAMD)

**Figure 6:**
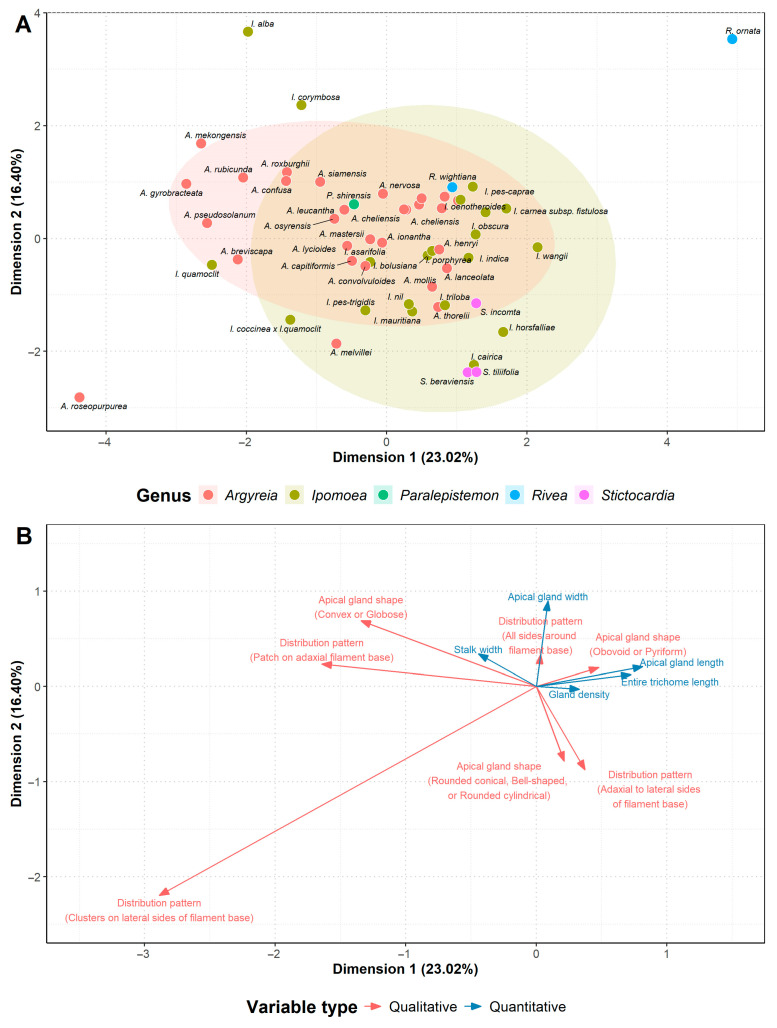
Results of factor analysis of mixed data (FAMD) conducted on species with only glandular staminal trichomes (group ii): (**A**) Scatter plot of examined species (dots). Each genus is shown by different colors with 95% confidence ellipses demonstrating variation found within the genus. Note that ellipses were not generated for *Paralepistemon*, *Rivea*, and *Stictocardia* due to insufficient sample sizes. (**B**) Loading plot of staminal trichome characters. Arrows indicate the direction of the character influencing species distribution in (**A**). Quantitative characters are shown in blue. Qualitative characters with character state in parenthesis are displayed in red.

The analysis of species in group (ii), the species with only glandular staminal trichomes, showed that the first two dimensions accounted for 39.42% of total variance (Table S2; Figure 6 and Figure S1). The 95% confidence ellipses of the genera in this group overlapped (Figure 6), with *Ipomoea* presenting the greatest variation and overlapping all other genera. Species of *Argyreia* and *Stictocardia* tended to cluster with other members within the same genus (Figure 6). *Rivea ornata* (Roxb.) Choisy were distinct compared with other genera, yet *R. wightiana* R.R. Mill was situated squarely among species of *Argyreia* and *Ipomoea*. Two quantitative characters and one qualitative character were identified to contribute strongly to the distribution of the species in the first dimension, i.e., apical gland length, entire trichome length, and apical gland shape (Figure S2A). There was one important character in the second dimension, which was apical gland width (Figure S2B).

**Figure 7:**
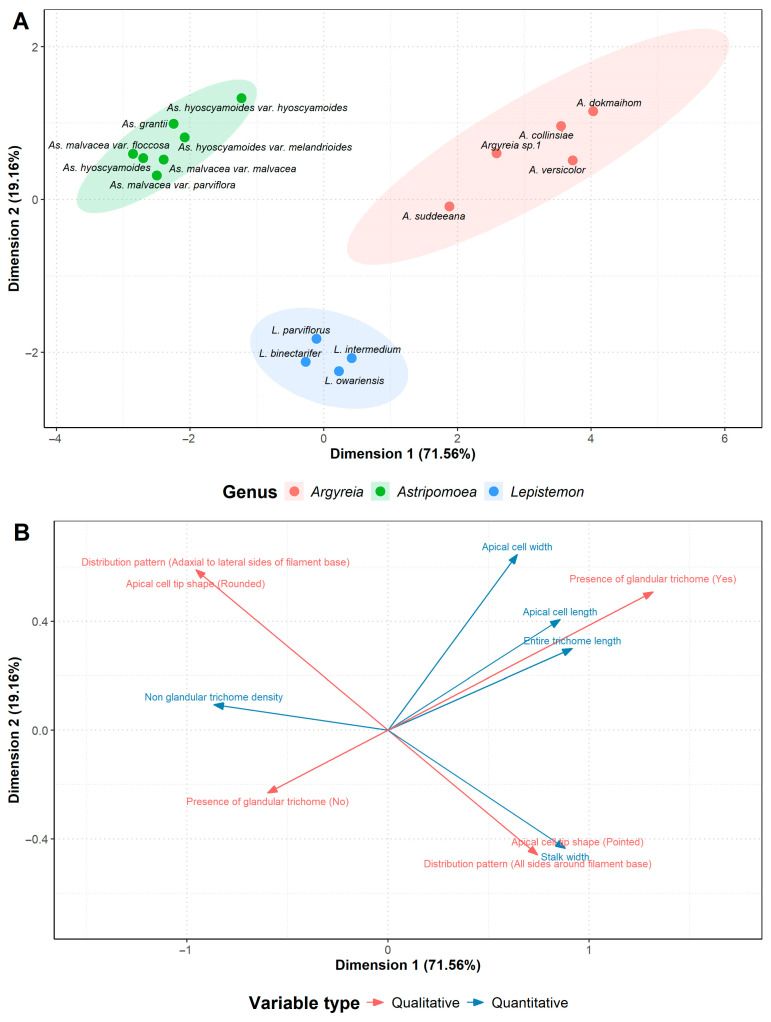
Results of factor analysis of mixed data (FAMD) conducted on species with nonglandular staminal trichomes (group iii): (**A**) Scatter plot of examined species (dots). Each genus is shown by different colors with 95% confidence ellipses demonstrating variation found within the genus. (**B**) Loading plot of staminal trichome characters. Arrows indicate the direction of the character influencing species distribution in (**A**). Quantitative characters are shown in blue. Qualitative characters with character state in parenthesis are displayed in red.

The analysis of species in group (iii), the species with nonglandular staminal trichomes, revealed that the first two dimensions accounted for 90.72% of the total variance (Table S3; Figure 7 and Figure S3). Species in this group clustered by genera without overlapping other genera (Figure 7). Four quantitative characters were identified as highly important characters in the first dimension, i.e., entire trichome length, stalk width, apical cell length, and nonglandular trichome density (Figure S4A). One quantitative character was found to contribute strongly to the second dimension, i.e., apical cell width (Figure S4B).

## 4. Discussion

Our work provides the first comprehensive investigation of staminal trichomes in the morning glory family, using a number of taxa sampled across genera in the tribe Ipomoeeae. The results of this study demonstrate that these plants possess diverse staminal trichome morphologies caused by ensembles of detailed structural elements. We utilized a more detailed approach in addition to the use of traditional terms to describe surface texture (e.g., pilose, villous, and hispid), which have been in use for over a century [5,10,11,12,28,48]. It is likely that the ‘soft’ features described in various literature reports refers to the long-stalked glandular trichomes observed in this study, while the ‘stiff’ features reported by such studies refer to the apical parts of the nonglandular trichomes.

The taxonomic value of staminal trichomes has traditionally been limited to their presence or absence, although the number of species without staminal trichomes was found to be very low in this study (only two species out of 73 taxa). The absence of staminal trichomes in these two species, *Argyreia adpressa* and *Ipomoea stenosiphon*, corresponds with their description in taxonomic literature [26]. However, literature describing the filament base as glabrous could be interpreted in several ways: staminal trichomes could be completely absent, present in small amounts (such as in *A. pseudosolanum* and *I. pes-tigridis* L.) [14,29], or hidden in positions difficult to observe (such as in *A. roseopurpurea*). This could easily lead to misunderstanding if observations were made without sufficient magnification [26].

Variation in staminal trichomes mostly stemmed from the different shapes of the apical cell, especially for glandular trichomes. Obovoid, pyriform, or rounded cylindrical glands were the most common shapes found in this study, and they were also previously portrayed in a few previous studies [5,6,7,18,27,29,49]. According to the FAMD, since the genera of group (ii) did not form distinct clusters due to the significant variation within *Ipomoea* that caused it to overlap with other genera, the high loading characters for this group were not able to differentiate between genera, even though significant differences were found among their quantitative characters. However, *Rivea* is likely to be the easiest genus to identify because three out of four high loading characters for this genus were significantly different from other genera in the group. In contrast, in group (iii), the high loading characters for this group, such as entire trichome length and stalk width, are highly useful for generic delimitation since the micromorphology of nonglandular staminal trichomes was distinctly different between genera.

*Argyreia* and *Ipomoea* were the genera represented by the most species in this study, in part because they are large genera and, for *Argyreia*, because Thailand is in the center of its geographical distribution and home to many species [35,50]. As these two genera are phylogenetically closely related [31,51], their morphology and micromorphology are typically quite similar, as also seen by the great variation in staminal trichome characters found in this study. The main difference between these genera is that some taxa of *Argyreia* possessed both glandular and nonglandular staminal trichomes, while all species of *Ipomoea* possessed only glandular trichomes.

The three species of *Stictocardia* examined in this study (*S. beraviensis* (Vatke) Hallier f., *S. tiliifolia*, and *S. incomta* (Hallier f.) Hallier f.) exhibited similar staminal trichome characters and could be discriminated from other genera by a combination of distribution pattern (adaxial to lateral sides of filament bases), apical gland shape (rounded cylindrical), and its significantly longer trichomes. While glandular staminal trichomes have been briefly mentioned in the descriptions of species in this genus [52], a study by Johnson [53] on two Australian *Stictocardia*, *S. tiliifolia* and *S. queenslandica* (Domin) R.W. Johnson, provided fine details regarding the staminal trichomes, including descriptions of the club-shaped to cylindrical apical cells, which is congruent with our results. Our findings also emphasize that micromorphology is valuable for the delimitation of this genus, in addition to the presence of black dots (prominent dark peltate glandular trichomes) on various parts of the plants [54,55].

*Paralepistemon* is a small genus in the family, consisting of only two species, and little is known about them [56,57]. The one species examined in this study, *P. shirensis* (Oliv.) Lejoly & Lisowski, was confirmed to have staminal trichomes, which were previously noted as glandular hairs in its first generic circumscription [57]. Most of the staminal trichome morphology of *Paralepistemon* resembles that of *Ipomoea*. However, the main characteristic that sets *Paralepistemon* apart is the enlarged base of the stamen, which takes on a scale-like shape, a feature specific to *Lepistemon* and *Lepistemonopsis* [57,58,59]. However, our study provided further evidence that the staminal trichomes in *Lepistemon* are of the nonglandular type, while those in *Paralepistemon* are of the glandular type. Although species of *Lepistemonopsis* could not be sampled in this study, the available information on the genus states that the staminal scale is glabrous [60], suggesting that staminal trichomes are possibly absent in *Lepistemonopsis* or, if present, that they would likely be smaller than those in *Lepistemon* and *Paralepistemon*.

*Rivea* is a small genus comprising three species [61], and two of them were included in our work, *R. wightiana* and *R. ornata*. Interestingly, while the staminal trichomes of *R. wightiana* were similar to those in *Argyreia* and *Ipomoea*, the trichomes of *R. ornata* were considerably larger than those of the other examined taxa in terms of entire trichome length and apical gland length and width. *Rivea* provides good examples of species without enlarged filament bases due to its narrow corolla tube, causing the staminal trichomes to appear to be distributed on the surface of the corolla tube as well. The distributions of staminal trichomes of all three species of *Rivea* are well-illustrated in a study by Staples [61].

Among the genera with nonglandular staminal trichomes, their trichome characteristics were distinctly different from each other. The staminal trichomes of *Astripomoea* were sessile and their apical cells had rounded tips, whereas those in *Argyreia* and *Lepistemon* were stalked, and their apical cells had pointed tips. The most important characteristic of *Astripomoea* is that it is the only genus in the tribe Ipomoeeae that possesses stellate hairs on vegetative parts [59,62], but its staminal trichomes, as confirmed in this study, are simple. The trichomes in *Argyreia* were found to be significantly longer than those in the other two genera. They were also found to be similar to the simple hairs occurring on vegetative organs [21,27,63], except that the staminal trichomes have a stalk. In *Lepistemon*, the stout stalk is more prominent than the short and slim apical cell, often leading the filament bases to be described or illustrated as papillose [8,14,59].

Our findings demonstrate that the general structure of staminal trichomes in the tribe Ipomoeeae is fairly consistent, as they are typically hair-like with an apical cell. The apical cells, specifically for the glandular trichomes, are typically obovoid to cylindrical. However, some species exhibited unique characteristics that may be potentially useful for identification. These characteristics are worth mentioning, in addition to those previously noted such as the trichomes in *Astripomoea* and *Lepistemon*. Nipple-like glands were found in *Argyreia roseopurpurea*, *A. lycioides* (Choisy) Traiperm & Rattanakr. (syn. *Blinkworthia lycioides* Choisy), and *A. convolvuloides* (Prain) Rattanakr. & Traiperm (syn. *B. convolvuloides* Prain) [18] (Figure 1B,H and Figure 3A,M). Multicellular apical glands were found in *Argyreia henryi* and *A. melvillei* (Figure 3Q). Stalks that enlarge toward the terminal end to subtend a wide apical gland were found in *Ipomoea alba* and *I. corymbosa* (L.) Roth. (syn. *Turbina corymbosa* (L.) Raf.). Moreover, the apical glands of *I. corymbosa* appeared with lobes (Figure 3C,D).

## 5. Conclusions

Our findings revealed that out of the 73 taxa examined across seven genera in the tribe Ipomoeeae, only two species, *Argyreia adpressa* and *Ipomoea stenosiphon*, lacked staminal trichomes. Glandular staminal trichomes were the predominant type in most examined taxa. *Ipomoea*, *Paralepistemon*, *Rivea*, and *Stictocardia* exclusively possessed glandular staminal trichomes. Most species of *Argyreia* had only glandular trichomes, although some species exhibited both glandular and nonglandular types. In contrast, *Astripomoea* and *Lepistemon* only possess nonglandular staminal trichomes. The appearance of staminal trichomes, whether glandular or nonglandular, was micromorphologically diverse in terms of shape, size, density, and distribution pattern. However, they consistently comprised two distinct parts, a stalk and an apical cell, except for the trichomes in *Astripomoea*, which were rather sessile. Genera with only glandular staminal trichomes exhibited variation that overlapped with those of other genera, with *Ipomoea* showing the greatest variation and overlapping with all other genera. However, genera with nonglandular staminal trichomes had distinct characteristics. Thus, the significant morphometric traits obtained from statistical analysis, such as entire trichome length and apical cell length and width, are highly applicable for the delimitation of genera with nonglandular staminal trichomes. Moreover, some taxa also demonstrated unique characteristics that could be useful for species delimitation, such as nipple-like glands (compared with the typical hair-like glands) and multicellular glands (compared with the typical unicellular glands). As staminal trichomes are widely present in various genera across the Convolvulaceae, this study provides a detailed approach to examining these taxonomically valuable microscale features. Our findings also emphasize that micromorphological and anatomical characters are equally important as other characters for taxonomic purposes.

## Figures and Tables

**Table 1:** Summary of staminal trichome details found in group (ii), the group of species with only glandular staminal trichomes.

Genus	Distribution Pattern	Apical Gland Shape	Entire Trichome Length (µm)	Stalk Width (µm)	Apical Gland Length (µm)	Apical Gland Width (µm)	Density (per mm^2^)
*Argyreia*	1, 2, 3, 4	1, 2, 3	496.26 ± 6.36	65.37 ± 0.35	101.59 ± 1.25	56.16 ± 0.39	33.58 ± 0.68
*Ipomoea*	3, 4	1, 2, 3	661.34 ± 9.25	50.85 ± 0.51	114.14 ± 2.1	56.5 ± 1.08	33.17 ± 1.65
*Paralepistemon*	4	1	509.87 ± 18.93	48.29 ± 2.01	83.89 ± 2.8	61.16 ± 1.38	68.00 ± 10.07
*Rivea*	4	2, 3	1490.3 ± 46.25	50.74 ± 1.21	337.79 ± 11.31	126.74 ± 2.28	49.09 ± 5.38
*Stictocardia*	3	2, 3	992.38 ± 26.11	46.24 ± 1.33	89.22 ± 3.51	30.84 ± 1.11	44.00 ± 4.34

Note: Quantitative characters are shown by mean ± SE. Numbers in distribution pattern column denote character states: 1, clusters on lateral sides of filament base; 2, patch on adaxial filament base; 3, adaxial to lateral sides of filament base; 4, all sides around filament base. Numbers in apical gland shape column denote character states: 1, convex or globose; 2, rounded conical, bell-shaped, or rounded cylindrical; 3, obovoid or pyriform.

**Table 2:** Summary of staminal trichome details found in group (iii), the group of species with nonglandular staminal trichomes.

Genus	Distribution Pattern	Presence of Glandular Trichome among Nonglandular Type	Apical Cell Tip Shape	Entire Trichome Length (µm)	Stalk Width (µm)	Apical Cell Length (µm)	Apical Cell Width (µm)
*Argyreia*	4	Yes	Pointed	1297.93 ± 29.8	69.3 ± 0.81	1169.52 ± 30.45	33.39 ± 0.42
*Astripomoea*	3	No	Rounded	252.95 ± 6.99	23.37 ± 0.46	252.95 ± 6.99	23.37 ± 0.46
*Lepistemon*	4	No	Pointed	380.70 ± 10.74	67.90 ± 2.29	243.59 ± 9.84	18.73 ± 0.73

Note: Quantitative characters are shown by mean ± SE. Numbers in distribution pattern column denote character states: 3, adaxial to lateral sides of filament base; 4, all sides around filament base.

## Data Availability

The original contributions presented in the study are included in the article/Supplementary Material, further inquiries can be directed to the corresponding author.

## Supplementary Materials

The following supporting information can be downloaded at https://www.mdpi.com/article/10.3390/plants13152050/s1, Figure S1: Scree plot of FAMD result of species with only glandular staminal trichomes; Figure S2: Percentage contribution of characters in the first two dimensions from results of FAMD of species with only glandular staminal trichomes (group ii); Figure S3: Scree plot of FAMD result of species with nonglandular staminal trichomes; Figure S4: Percentage contribution of characters in the first two dimensions from results of FAMD of species with nonglandular staminal trichomes (group iii); Table S1: Specimen information of plant materials used in this study; Table S2: Loading values of staminal trichome characters and eigen analysis of the first two dimensions from FAMD results of species with only glandular staminal trichomes (group ii); Table S3: Loading values of staminal trichome characters and eigen analysis of the first two dimensions from FAMD results of species with nonglandular staminal trichomes (group iii); Table S4: Summary of staminal trichome details in examined accession no.
