# Supplementary material for: Intergeneric Comparison of Staminal Trichomes in the Tribe Ipomoeeae (Convolvulaceae)
Source: Plants (Basel). 2024 Jul 25;13(15):2050. doi: 10.3390/plants13152050 (PMC11314094; doi:10.3390/plants13152050)
Supplement: Supplementary file 1 [file plants-13-02050-s001.zip › Figure.pdf]

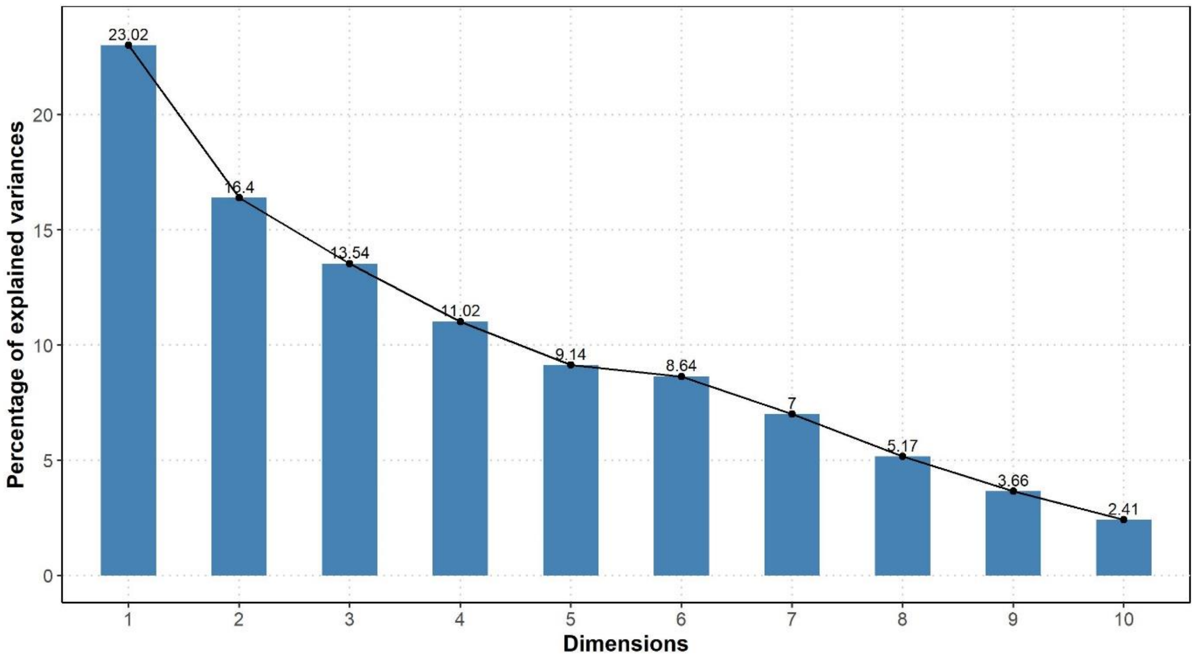

Figure S1. Scree plot of FAMD result of species with only glandular staminal trichomes. Bars, dots, and numbers indicate percentage of explained variance in each dimension.

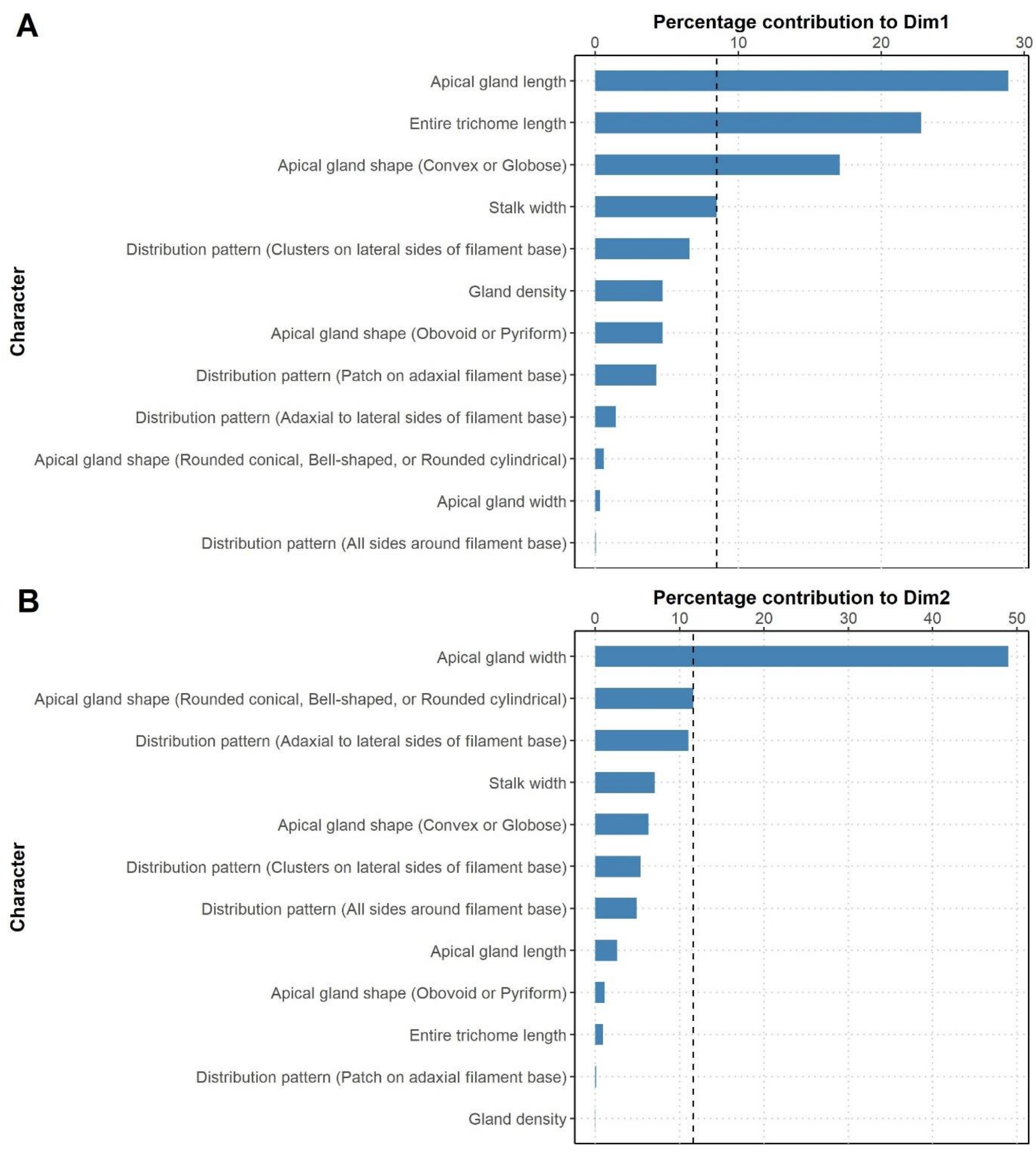

Figure S2. Percentage contribution of characters in the first two dimensions from results of FAMD of species with only glandular staminal trichomes (group ii). (A) Percentage contribution in the first dimension. (B) Percentage contribution in the second dimension. Dashed lines indicate cut-off values used to identify the high loading characters. Note that qualitative characters are listed by character names followed by character states in parenthesis.

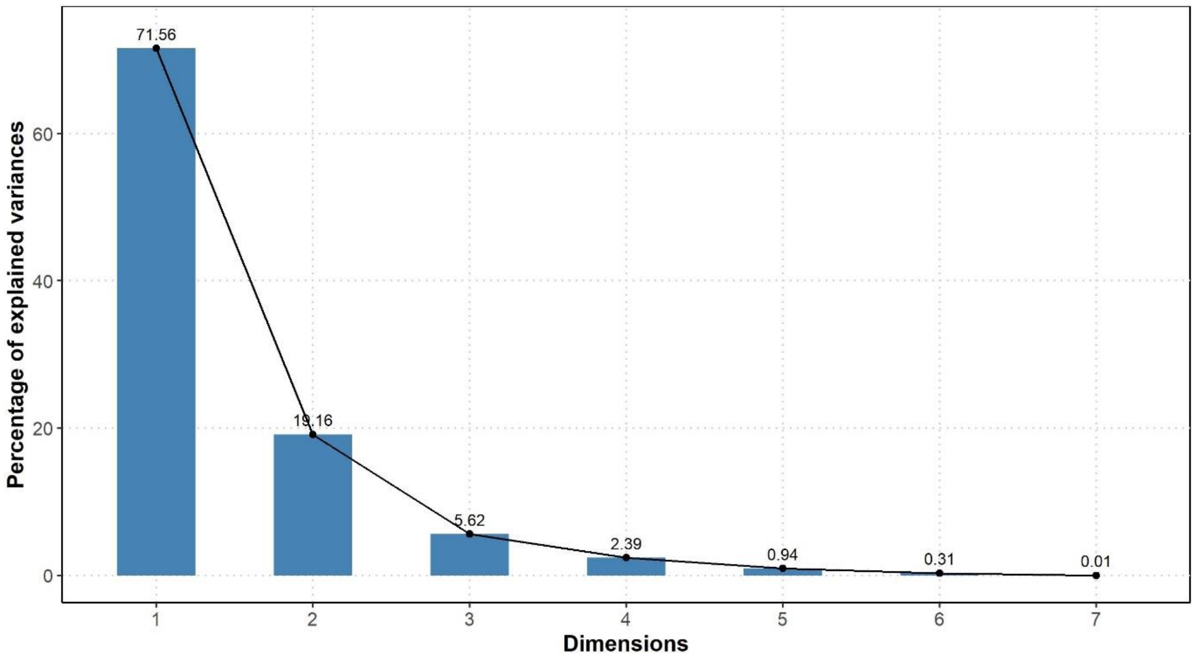

Figure S3. Scree plot of FAMD result of species with non-glandular staminal trichomes. Bars, dots, and numbers indicate percentage of explained variance in each dimension.

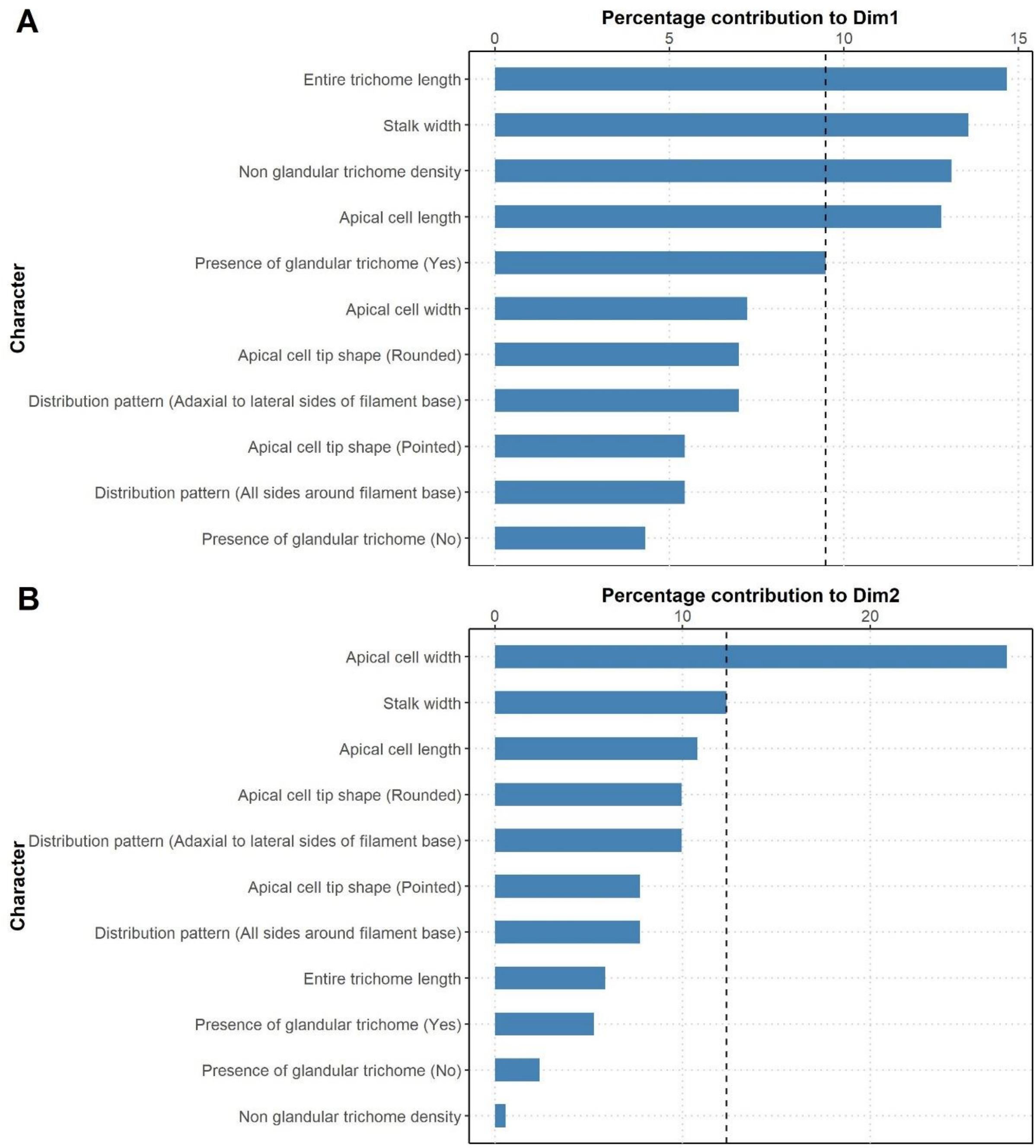

Figure S4. Percentage contribution of characters in the first two dimensions from results of FAMD of species with non-glandular staminal trichomes (group iii). (A) Percentage contribution in the first dimension. (B) Percentage contribution in the second dimension. Dashed lines indicate cut-off values used to identify the high loading characters. Note that qualitative characters are listed by character names followed by character states in parenthesis.
