# Supplementary material for: Intergeneric Comparison of Staminal Trichomes in the Tribe Ipomoeeae (Convolvulaceae)
Source: Plants (Basel). 2024 Jul 25;13(15):2050. doi: 10.3390/plants13152050 (PMC11314094; doi:10.3390/plants13152050)
Supplement: Supplementary file 1 [file plants-13-02050-s001.zip › Table S2-S3.pdf]

Table S2. Loading values of staminal trichome characters and eigen analysis of the first two dimensions from FAMD results of species with only glandular staminal trichomes (group ii)

**Character types**	**Characters**	**Dimension 1**	**Dimension 2**
Quality	Distribution pattern	0.283	0.351
Quality	Apical gland shape	0.516	0.312
Quantity	Entire trichome length	0.525	0.015
Quantity	Stalk width	0.196	0.115
Quantity	Apical gland length	0.665	0.043
Quantity	Apical gland width	0.008	0.803
Quantity	Gland density	0.109	0.001
	**Eigenvalue**	**2.30**	**1.64**
	**Cumulative % of eigenvalue**	**23.02**	**39.42**

Table S3. Loading values of staminal trichome characters and eigen analysis of the first two dimensions from FAMD results of species with non-glandular staminal trichomes (group iii)

**Character types**	**Characters**	**Dimension 1**	**Dimension 2**
Quality	Distribution pattern	0.712	0.271
Quality	The presence of glandular trichome	0.789	0.118
Quality	Apical cell tip shape	0.712	0.271
Quantity	Entire trichome length	0.840	0.090
Quantity	Stalk width	0.777	0.189
Quantity	Apical cell length	0.732	0.166
Quantity	Apical cell width	0.414	0.418
Quantity	Non-glandular trichome density	0.749	0.009
	**Eigenvalue**	**5.72**	**1.53**
	**Cumulative % of eigenvalue**	**71.56**	**90.72**
